# Cancer cell membrane-coated mesoporous silica loaded with superparamagnetic ferroferric oxide and Paclitaxel for the combination of Chemo/Magnetocaloric therapy on MDA-MB-231 cells

**DOI:** 10.1038/s41598-019-51029-8

**Published:** 2019-10-09

**Authors:** Defu Cai, Likun Liu, Cuiyan Han, Xiaoxing Ma, Jiayi Qian, Jianwen Zhou, Wenquan Zhu

**Affiliations:** 1 Institute of Medicine and Drug Research, Qiqihar Medical University, Qiqihar, 161006 China; 2 College of Pharmacy, Qiqihar Medical University, Qiqihar, 161006 China

**Keywords:** Oral cancer, Drug discovery

## Abstract

To effectively inhibit the growth of breast cancer cells (MDA-MB-231 cells) by the combination method of chemotherapy and magnetic hyperthermia, we fabricated a biomimetic drug delivery (CSiFePNs) system composed of mesoporous silica nanoparticles (MSNs) containing superparamagnetic ferroferric oxide and Paclitaxel (PTX) coated with MDA-MB-231 cell membranes (CMs). In the *in vitro* cytotoxicity tests, the MDA-MB-231 cells incubated with CSiFePNs obtained IC_50_ value of 0.8 μgL^−1^, 3.5-fold higher than that of SiFePNs. The combination method of chemotherapy and magnetic hyperthermia can effectively inhibit the growth of MDA-MB-231 cells.

## Introduction

Breast cancer is a disease that seriously threatens the health of people in the world^1–5^. In clinic, the chemotherapy is still the standard method for breast cancer treatment. Severe side effects and poor delivery efficiency of chemotherapy agents severely limit their therapeutic efficacy^6–10^. With the changes in the understanding of biological behaviors of breast cancer and the renewal of treatment concepts, the treatment of cancer has entered the era of comprehensive treatment, forming a treatment mode that emphasizes combination therapy of breast cancer, such as magnetic hyperthermia and photodynamic therapy^11–15^.

In recent years, magnetic hyperthermia therapy has been widely used in the cancer treatment *in vivo* because of a few excellent advantages such as heat-induced properties, chemical stability, targeting ability and so on^13,16–21^. The magnetic hyperthermia technique can avoid unnecessary harm to human health while killing tumor cells. It is because of that the energy of the alternating magnetic field is absorbed only by paramagnetic particles and is absorbed merely by normal human tissues^22,23^. When paramagnetic particles are exposed to an alternating magnetic field, they oscillate with the applied alternating magnetic field. The magnetic field energy are converted into heat due to energy dissipation from paramagnetic particles. High temperatures also interfere with the regulation of biological processes in tumor cells, such as proliferation and metabolism. Moreover, tumor cells exposed to temperatures above 41–46 °C can be induced to necrosis or apoptosis^24,25^.

Although *in situ* local tumors can be successfully removed by magnetic nanoparticles, they may induce human normal cells to produce immune rejection and easily penetrate and escape from the tumor tissue due to their exogenous^26,27^. Therefore, the ideal magnetic nanoparticles should have immune escape and tumor accumulation abilities for the purpose of treating tumors. In recent years, many studies have been aimed at avoiding immune clearance and improving the recognition ability by surface modification with targeting ligands^28–30^. However, active targeting effects are severely affected by the receptor expression density and number of target sites. Surface-modified substances may also activate the immune system and cause the efflux of nanoparticles to some extent. Therefore, magnetic hyperthermia technology is often restricted in its application.

The emergence of cell membrane carriers have opened up a new path for the development of nanotechnology^31–34^. Compared with traditional pharmaceutical carriers, such as liposomes and polymer nanoparticles, cell membrane carriers offer some significant advantages. In recent years, red blood cell membranes have been widely studied as typical cell membrane carriers. Red blood cell membrane as human endogenous substances can prevent the encapsulated nanoparticles from being cleared by the body’s immune system and increased circulation time of nanoparticles in the body^35,36^. Although the red blood cell membranes exhibit excellent performance as a carrier, they cause the nanoparticles to reduce their targeting ability. Excitingly, studies have shown that the structure of multiple membranes confers immune escape and homotypic binding characteristics on cancer cells^37^. Mesoporous silica is often used as a carrier for chemotherapeutic drugs, such as doxorubicin or paclitaxel. In recent years, more and more research have focused on the application of mesoporous silica to chemotherapy in combination with other treatments to treat cancer, such as magnetothermal therapy, photothermal therapy and radiation therapy. For example, Zhengfang Tian, *et al*., developed a potential chemo- and magnetic hyperthermia therapeutic mesoporous silica nanoparticles, which showed a synergistic effect hyperthermia that combined chemotherapy and magnetic hyperthermia therapy^38^. Cuilian Tao, *et al*., prepared DNA-capped Fe_3_O_4_/SiO_2_ magnetic mesoporous silica nanoparticles for controlling drug Release by regulating the temperature and perform magnetic hyperthermia^39^. Many scholars use mesoporous silica as a carrier to treat tumors by combining chemotherapy with magnetocaloric therapy^40,41^. Inspired by the homotypic binding characteristics of cancer cells membranes during the contact of the tumor cell membrane, we expect to establish a breast cancer cell-specific accumulation preparation with bionic properties, using the breast cancer cell membrane as a carrier, which can load superparamagnetic ferroferric oxide nanoparticles and drugs into target breast cancer cells and eliminate them by the combination method of magnetic hyperthermia and chemotherapy.

Herein, we designed a cancer cell membrane-coated mesoporous silica nanoparticles with homotypic targeting ability and magnetic hyperthermia properties. Firstly, PTX and superparamagnetic ferroferric oxide were both loaded into mesoporous silica nanoparticles (SiFePNs). Secondly, MDA-MB-231 cell membranes were extracted from MDA-MB-231 cells. At last, SiFePNs were coated with the CMs by extrusion method (CSiFePNs). It was hypothesized that CSiFePNs could selectively accumulate in homologous tumor cells, it generated heat under the action of an alternating magnetic field and promoted tumor cell apoptosis together with chemotherapeutic drugs. It was expected that the combination method of chemotherapy and magnetic hyperthermia could be used to enhance the inhibition of growth for MDA-MB-231 cells.

## Experimental

### Materials

Superparamagnetic ferroferric oxide (Fe_3_O_4_, 10–20 nm) were obtained from XFNano Co., LTD (Nanjing, China). Pluronic block co-polymer F127 was kindly donated by BASF. Tetraethyl orthosilicate (TEOS), Hexadecyl trimethyl ammonium Bromide(CTAB), hydrochloric acid, ethanol and MgCl_2_ were purchased from Kaitong chemical Reagent Co. Ltd. (Tianjin, China). Paclitaxel (PTX) was obtained from ycgcphar Co. Ltd (Wuhan, China). PBS buffer, Tris, EDTA-free protease inhibitor, formazan, dimethyl-sulfoxide (DMSO), Nile red and Hoechst 33342 was obtained from Sigma-Aldrich (St. Louis, MO, USA). All chemicals were in accordance with the requirements of analytical/spectroscopic/HPLC grade. Deionized water was obtained by ion exchange.

### Preparation of CSiFePNs

First, the mesoporous silica nanoparticles (MSN) contained Fe_3_O_4_ nanoparticles were prepared by the published method^42^. 2.0 g of Fe_3_O_4_ nanoparticles, 3.0 g of CTAB, 20 mL of ethanol and 80 mL distilled water were mixed under gently stirring for 20 min at 25 °C. Then, 3.5 mL of TEOS was added drop by drop to the mixture under stirring. After the solution was stirred for 1.5 h, Finally, the obtained mixture was centrifuged to remove the supernatant. The recipitate was washed with distilled water and ethanol for three times. The solid white substance was dried under vacuum for 24 h. The products were labeled as SiFeNs. Secondly, PTX was incorporated into SiFeNs by a solvent deposition method^43^. In detail, PTX was dissolved in methanol to obtain a concentrated solution (2 mg/mL), and then 100 mg of SiFeNs was added into the drug solution to obtain a mixture. The mixture was still gently stirring for 48 h at room temperature in a closed container to complete the adsorption equilibrium operation. Finally, the products were dried at 40 °C to remove organic solvent. The drug-loaded samples were labeled SiFePNs. Thirdly, the MDA-MB-231 cell membrane vesicles (CMs) as the outer shells were prepared[38]. In brief, the MDA-MB-231 cells were washed with PBS for three times and suspended in buffer the hypertonic Tris buffer (10 mM Tris, 10 mM MgCl_2_ and 1 x EDTA-free protease inhibitor, pH = 7.4) at 4 °C for 1 h. Then, the cell suspension was homogenized in a tissue homogenizer for 1 min and centrifuged at 500 rmp for 10 min. The supernatants were centrifuged at 10000 rmp for 10 min and 35000 rmp for 1 h (Hitachi CR22G, Japan) to get the cell pellets. After the supernatants were removed, the cell membranes deposited at the bottom was resuspended in PBS solution and sonicated for 5 s by a sonic cell pulverizer (Biosafer 900–92, China). Finally, the obtained pellets was extruded through 400 nm polycarbonate membranes for about 11 times by a extruder (Morgec LE-15, China). The resulting CMs were collected. To obtain nanoparticles encapsulated by MDA-MB-231 cell membrane, the CMs were sequentially extruded through a 400 nm and a 200 nm polycarbonate membrane for 15 times. Afterwards, the SiFePNs were co-extruding with CMs at mass ratio of 2.0 (SiFePNs: CMs) for 12 times. At last the resulting nanoparticles were collected to obtain CSiFePNs.

## Characterization Techniques

### TEM study

The internal structure of the samples was evaluated by TEM (CSIS EM-208S, USA). The minute quantities of samples were fixed on the copper network to be characterized.

### Particle size and potential analysis

The zeta potential and particle size of MFeNs, SiFePNs and CSiFePNs were studied by DLS analyzer (Malvern Zetasizer, UK).

### Nitrogen adsorption-desorption measurement

Nitrogen adsorption-desorption analysis was carried out by an adsorption analyzer (VSorb 2800 P, China). The physically adsorbed water of samples was volatilized in the oven at 55 °C for 24 h. Then, the samples were placed in liquid nitrogen. At last, the specific surface area and the pore volume of the samples were measured by a surface area analyzer.

### Magnetization study

Magnetization curves were drawn and analyzed by a vibrating magnetometer (Lake Shore 7410, USA) at 300 K. The hysteresis of magnetization was produced by regulating H from + 10,000 Oe to −10,000 Oe.

### FT-IR study

The FT-IR spectrometer (Thermo Nicolet 380, USA) was used to examine the chemical bonding and functional groups of samples. The FT-IR measurement was carried out by using the potassium bromide (KBr) tableting method. KBr was thoroughly ground in an agate mortar in advance, and then dried in an oven at 110–150 °C for about 48 hours. The obtained KBr was placed in a desiccator for storage. Before tableting, 200–300 mg of KBr from the desiccator and 1–1.5 mg of stone samples were thoroughly ground in an agate mortar. Then both of them were put in an oven at 100 °C for 5 minutes. At last the samples were continuously ground for about 30 seconds, with the spectral resolution of 4 cm^−1^. The samples were mixed with potassium bromide according to a certain ratio of 1–2:200, and then the mixture was pressed into a round cake and characterized in the scanning range of 400 cm^−1^–3500 cm^−1^.

### Raman spectra analysis

To further study the surface material of the sample, the Raman Imaging Microscope (Thermo U-LH100L-3, USA) was used to study functional groups of samples. After the slide was wrapped in tin foil, the samples were coated in foil and characterized in the scanning range of 50 cm^−1^–3300 cm^−1^. The scanning was completed by the mode of area sweep. The laser wavelength was set to 780 nm. The spectral resolution was 5 cm^−1^. The excitation power was 24 mW. The integration time was 0.2 s. The number of scans was 20 times. The detector was a TE-cooled electron-multiplying CCD (EMCCD). A 10 × long working distance microscope objective and a 50 μm confocal pinhole DXR780 full range grating (400line/mm) were both used.

### *In vitro* magnetic hyperthermia study

The alternating magnetic field generator (Litian CT-100, China) was used to study the heating capacity of CSiFePNs in AMF. The CSiFePNs solutions with a range of concentrations (20, 40, 60, 80 and 100 mg/ml) were placed in an alternating magnetic field generator, respectively. The temperature data were obtained by a thermometer at the fixed time points (5, 10, 15, 20, 25, 30, 35, 40, 45, 50, 55 and 60 min). Moreover, different current intensities (100 W, 200 W and 300 W) were also regulated to study the impact of current intensity on the heating capacity of CSiFePNs in AMF. In order to assess the release behavior of drugs in AMF, the solution of PTX, SiFePNs and CSiFePNs (5 mL, at [PTX] = 1.6 mg/mL) was placed in dialysis bags (molecular weight 100–500), which was immersed in 50 mL of PBS. The added samples were gently stirred at 25 °C and performed for 1 h in AMF (60 Gs). The drug release profiles were obtained at fixed intervals (5, 10, 15, 20, 30, 45 and 60 min).

### *In vitro* cytotoxicity assay

The *in vitro* cytotoxicity of MFeNs, SiFePNs, and CSiFePNs in MDA-MB-231 cells was measured by an MTT assay. The MDA-MB-231 cells were seeded in 96-well plates at a density of 5 × 10^3^ cells per well and incubated in medium (100 mL) for 24 hours to complete cell attachment. Sample solutions (MFeNs, SiFePNs or CSiFePNs) with a final concentration of 0.60 mg Fe/mL per well were added to replace the original medium, respectively. Subsequently, cells in the experimental group were placed in an alternating magnetic field and the cells in the control group were placed out of AMF. After 24 hours of incubation, 50 mL of MTT (1 mg/mL) was added into the well and the cells were continued to be incubated for 4 hours. Formazan was extracted by DMSO and a microplate reader (ThermoFisher Multiskan Spectrum, USA) was used to measure the absorbance at 540 nm. To study the safety of MSN and Fe_3_O_4_ on cells, the same *in vitro* cytotoxicity test as above was carried out. MSN and Fe_3_O_4_ with a series of concentrations were incubated with cells for 24 hours. The cell viability was also measured by the MTT assay.

### Cellular uptake

Flow cytometry was used to quantitatively analyze the uptake of PTX by MDA-MB-231 cells. The cells were evenly spread in 6-well plates at 2.0 × 10^5^ cells per well. After 24 hours of culture, the mediums containing PTX, SiFePNs or CSiFePNs (PTXs were all stained with Nile red) were added, respectively. After 1, 2, 4 hours of incubation, cells were washed with the PBS solution. Then, the cells were digested by trypsin and centrifuged at 1000 rpm for 5 min to separate cells from the supernatant. An appropriate amount of PBS solution was added to suspend cells. Finally, 200 μL of PBS solution was added to blow the cells into a cell suspension, which was detected and analyzed using a flow cytometer. The average fluorescence intensity of 2.0 × 10^5^ cell was measured using a flow cytometer.

### Laser confocal microscopy study

Microscope slides in 24-well plates (5 × 10^4^ cells per well) were used to culture MDA-MB-231 cells for 24 hours. MDA-MB-231 cells all adhering to the slides were used for subsequent experiments. Then the SiFePNs and CSiFePNs at a Coumarin-6 concentration of 100 ng/mL were added on the MDA-MB-231 cells. The culture medium was removed after 2 h of cultivation and ice-cold PBS was used to wash the MDA-MB-231 cells for three times. All the MDA-MB-231 cells were fixed with 4% paraformaldehyde solution for 25 min, followed by cell nuclei staining by Hoechst 33258 (10 ug/ml) for 20 min. The stained cells were washed with PBS for 3 times. Finally, a LSM710 laser confocal microscope (Zeiss, Germany) was used to capture the fluorescent images of the cells.

## Results and Discussion

### Preparation and characterization of CSiFePNs

**Figure 1:**
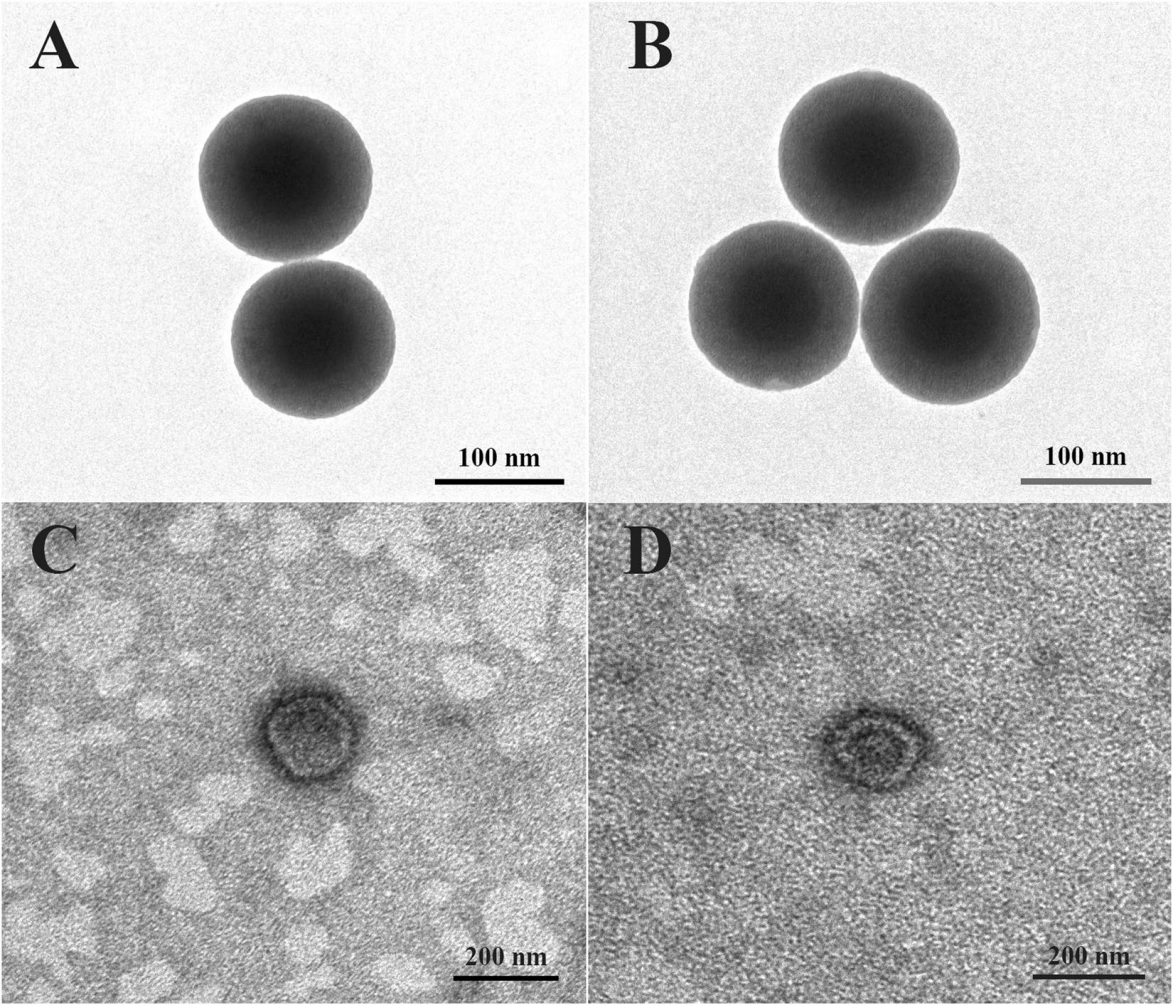
TEM images of (**A**) MSN, (**B**) SiFePNs, (**C**) CMs and (**D**) CSiFePNs.

**Figure 2:**
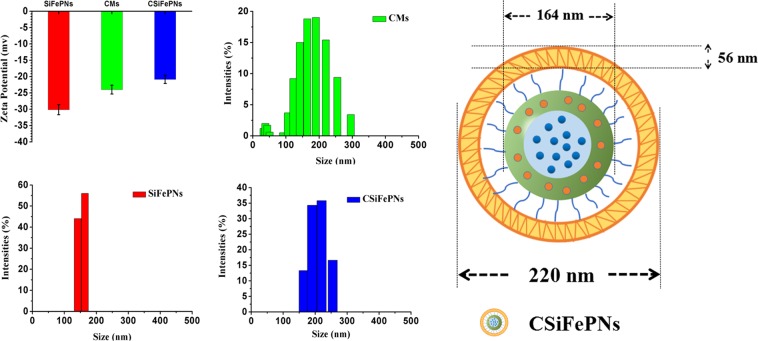
Particle size and zeta potential of the CMs, SiFePNs, and CSiFePNs.

CMs@MSN/Fe_3_O_4_/PTX nanoparticles (CSiFePNs) were thus synthesized by the following procedure. Firstly, MSN/Fe_3_O_4_ was synthesized by a self-assembly method, in which Fe_3_O_4_ was located in the core of MSN. In addition, the shell portion of MSN was used to load PTX. As can be seen from Fig. 1A, MSN shows a clear core-shell structure and spherical morphology with mesoporous channels. In addition, SiFePNs were also prepared by loading Fe_3_O_4_ and PTX into MSN. MSN demonstrates excellent mesoporous framework and drug loading capacity. Secondly, SiFePNs were coated with CMs, which were extracted from MDA-MB-231 cells’ membrane. As shown in Fig. 1D, the cell membranes were successfully coated around SiFePNs. As seen in Fig. 2, after membrane coating, the average size of CSiFePNs increases from 164 nm (PDI 0.04) to 220 nm (PDI 0.07), which may be due to the thickness of CMs. Moreover, the surface charge of CSiFePNs (−20.88 ± 0.4 mV) is the negative, the same as that of CMs. This further demonstrates that the CSiFePNs encapsulated by CMs exhibit the same properties as the cell membrane surface. The average size and zeta-potential both demonstrate that SiFePNs were successfully coated by CMs. As can be seen from Fig. S1, the H2 hysteresis loops of MSN belongs to the IV isotherms of the IUPAC classification, confirming the typical mesoporous structure of MSN. After Fe_3_O_4_ and PTX were loaded into MSN, the adsorption curve and the desorption curve of the isotherm are almost coincident, indicating that the pores of MSNs are filled with Fe_3_O_4_ and PTX. The pore volume (Vt) and surface area (S_BET_) data are shown in Table 1. The pore volume (0.25 cm^3^/g) and surface area (218.34 m^2^/g) of SiFePNs are significantly reduced compared with those (1.19 cm^3^/g and 845.93 m^2^/g) of MSN. This may be because the pore space is occupied by the drugs and Fe_3_O_4_. Fig. S2 shows the magnetization curve of CSiFePNs. CSiFePNs exhibit superparamagnetism with negligible hysteresis loops in low field, indicating the magnetic property of CSiFePNs loaded with Fe_3_O_4_.

**Table 1:** Results of nitrogen adsorption study.

Samples	S_BET_ (m^2^/g)	V_t_ (cm^3^/g)
MSN	845.93	1.19
SiFePNs	218.34	0.25

### FT-IR study

**Figure 3:**
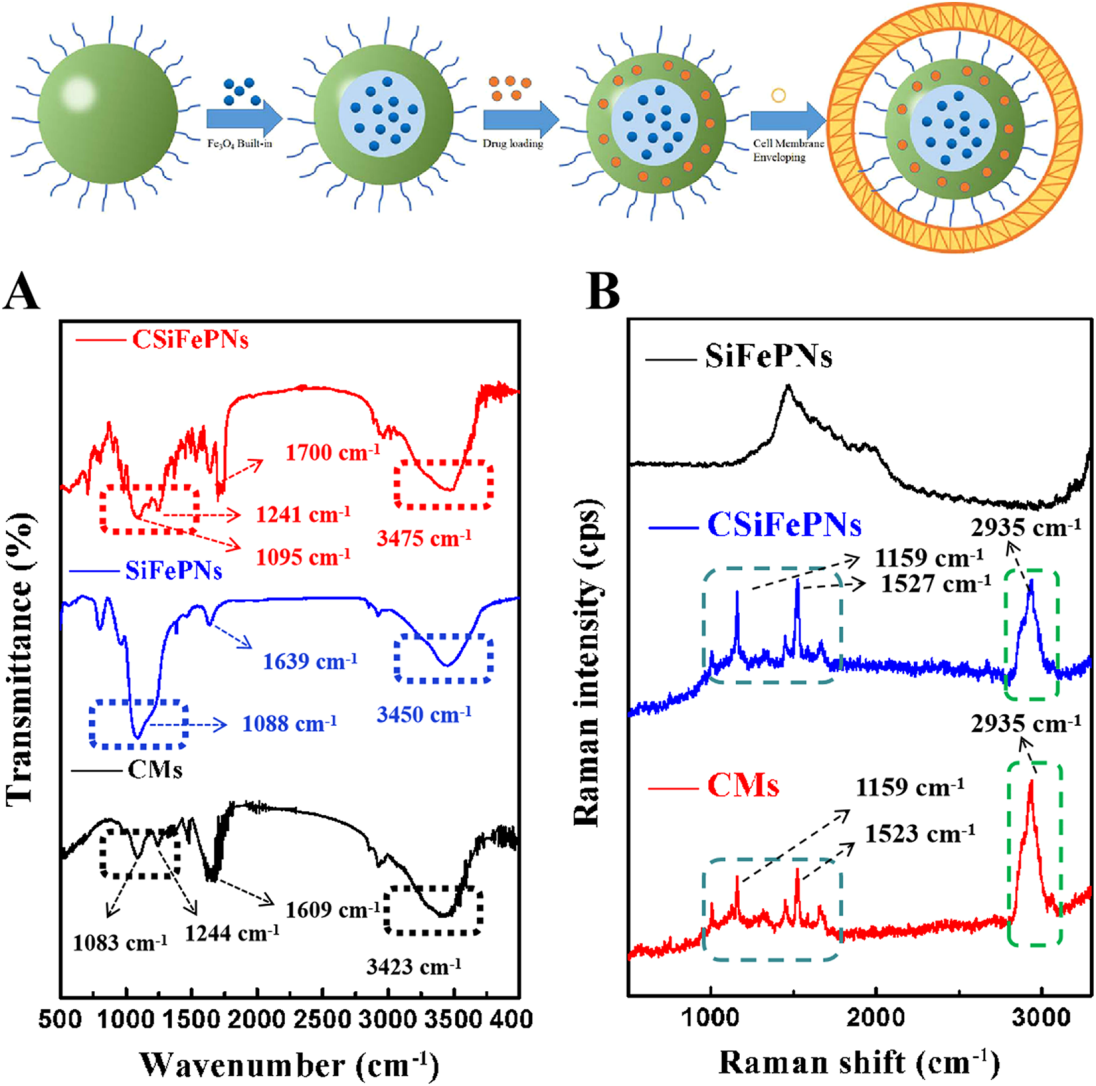
FTIR spectra of the CMs, SiFePNs, and CSiFePNs (**A**) and Raman spectra of the CMs, SiFePNs, and CSiFePNs (**B**).

FT-IR spectra were performed to confirm the process for preparing CSiFePNs. The FT-IR spectra of samples (CMs, SiFePNs and CSiFePNs) are shown in Fig. 3A. The SiFePNs show wide adsorption bands in the range of 3750 cm^−1^–3000 cm^−1^ due to the silanol groups. After SiFePNs were internalized in MDA-MB-231 cell membranes, the characteristic peaks of embedded particles are all weakened or disappeared. For example, the strong absorption peak (1088 cm^−1^) of SiFePNs is weakened. Moreover, the FT-IR spectra of CSiFePNs are significantly similar to that of the cell membrane for CMs. Two weaker absorption peaks (1244 cm^−1^ and 1083 cm^−1^) of CMs still exist in the FT-IR spectra of CSiFePNs (1241 cm^−1^ and 1095 cm^−1^). The FTIR spectral variation between CSiFePNs and SiFePNs at 2950 cm^−1^ in Fig. 3A can be clearly seen. CSiFePNs have an absorption peak at 2950 cm^−1^, whereas SiFePNs do not have this absorption peak. This absorption peak may be caused by the C-H bands’ stretching vibration of the methyl group in the cell membrane phospholipid. This result illustrates the presence of cell membranes on the surface of CSiFePNs. Thus, FT-IR spectra results show that MDA-MB-231 cell membranes successfully covered on the surface of SiFePNs.

### Raman spectra study

To further confirm the process for preparing CSiFePNs, Raman spectra method was used to characterize the structure of the samples (CMs, SiFePNs and CSiFePNs). As shown in Fig. 3B, there is a relatively blunt peak with a maximum at 1703 cm^−1^ for SiFePNs, which is the characteristic peak of SiFePNs. However, CMs show two sharp peaks at 1159 cm^−1^ and 1523 cm^−1^, respectively. CMs show strong peaks at 2935 cm^−1^, but SiFePNs do not have any peaks at this corresponding position. This indicates that the characteristic absorption peak of CMs is different from that of SiFePNs. The following reasons can be used to explain this phenomenon. The main components of CMs are phospholipids and cholesterol^44^. Conversely, the surface components of SiFePNs are mainly silica. Furthermore, the two sharp peaks at 1159 cm^−1^ and 1527 cm^−1^ also exist in the Raman spectra of CSiFePNs. The absorption peak of SiFePNs is covered by that of CMs. Moreover, CSiFePNs show the same peak as CMs at 2935 cm^−1^. This is a powerful proof that SiFePNs were completely internalized by CMs. Raman spectra results also show that CSiFePNs were successfully prepared.

### *In vitro* magnetocaloric characteristics study

**Figure 4:**
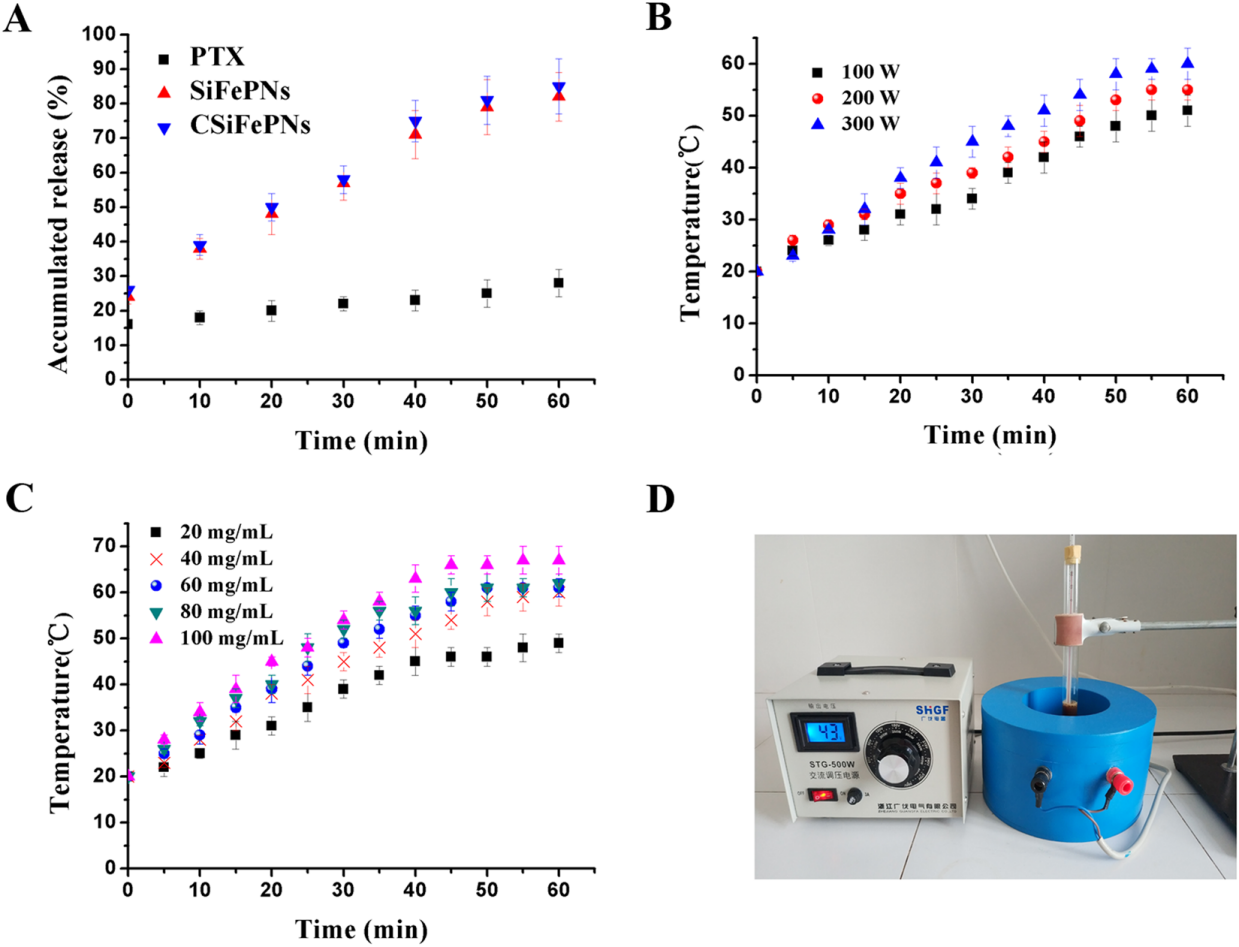
(**A**) *In vitro* release of PTX from CMs, SiFePNs, and CSiFePNs in ACMF. (**B**) The heating temperature of CSiFePNs under the alternating magnetic field magnetization of different current intensities after 60 min incubation. (**C**) The heating temperature of CSiFePNs with different concentrations under the alternating magnetic field magnetization after 60 min incubation. (**D**) Experimental device picture of alternating magnetic field generator.

*In vitro* magnetothermal conversion tests were used to evaluate the impact of magnetic field strength and solution particle concentration on the magnetocaloric effect. As shown in Fig. 4A, the temperature of CSiFePNs with different concentrations of 20 mg/ml, 40 mg/ml, 60 mg/ml, 80 mg/ml and 100 mg/ml in AMF within 60 min reaches 49 °C, 60 °C, 61 °C, 62 °C and 67 °C, respectively. Hence, it is concluded that magnetothermal conversion efficiency is influenced by the concentrations of CSiFePNs. This can be explained as follows. The superparamagnetic particles undergo a physical effect of converting magnetic energy into thermal energy under the action in AMF. The number of superparamagnetic particles determines the level of thermal energy. Additionally, the alternating magnetic field strength also has a certain influence on the magneto-thermal conversion effect. The temperatures of CSiFePNs solutions in AMF within 60 min follow the sequence: 60 °C(300 W) > 55 °C(200 W) > 51 °C(100 W). It can be concluded that the alternating magnetic field current intensity and the concentrations of CSiFePNs both have significant impacts on temperature changes. Furthermore, the process of magnetocaloric conversion can be controlled by regulating these above two factors.

### Cellular uptake of CSiFePNs

**Figure 5:**
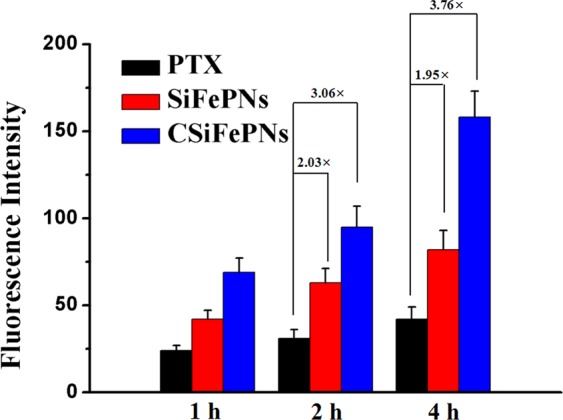
Fluorescence intensity of PTX, SiFePNs and CSiFePNs measured by flow cytometry after 1, 2, or 4 h incubation, respectively.

Cellular uptake test was used to evaluate the cellular uptake behaviors of CSiFePNs. As shown in Fig. 5, the fluorescence intensity of CSiFePNs is time-dependent. The fluorescence intensity of CSiFePNs in AMF after incubation for 4 hours is about 3.76-fold higher than that of PTX. Moreover, the fluorescence intensity of CSiFePNs is much higher than that of SiFePNs, indicating that the cell membrane coating significantly enhanced cellular uptake of SiFePNs. This is because the homotypic targeting ability produced by the retained cell membrane surface proteins makes them more easily be taken up by homologous cells. CMs can reduce the immunophagocytosis of nanoparticles by other tissues, which is due to the CD47 membrane protein^45,46^. CD47, a transmembrane protein on the surface of cell membranes, regulates immunity. Studies have shown that attaching CD47 to the surface of nanoparticles can significantly reduce their phagocytosis by macrophages^47^. The cell membrane completely encapsulates SiFePNs, and the CD47 protein abundantly expressed on the membrane can be recognized by macrophages from being phagocytized, which reduces the immune-related response of the body^48^. In addition, the fluorescence intensity of cells cultivated with CSiFePNs under the stimulation of AMF is obviously higher than that of CSiFePNs without induction of AMF (Fig. 5B). This is because the magnetocaloric effect of CSiFePNs induced by AMF leads to an increase in temperature, which destroys the fluidity of the cell membrane and accelerates the fusion of cells and CSiFePNs. In summary, the MDA-MB-231 cell membranes coated with SiFePNs increase the uptake of SiFePNs by homologous cells. The above results together indicate that CSiFePNs achieve the purpose of enhancing the cellular uptake ability of PTX by cell membranes coating.

### *In vitro* cytotoxicity

**Figure 6:**
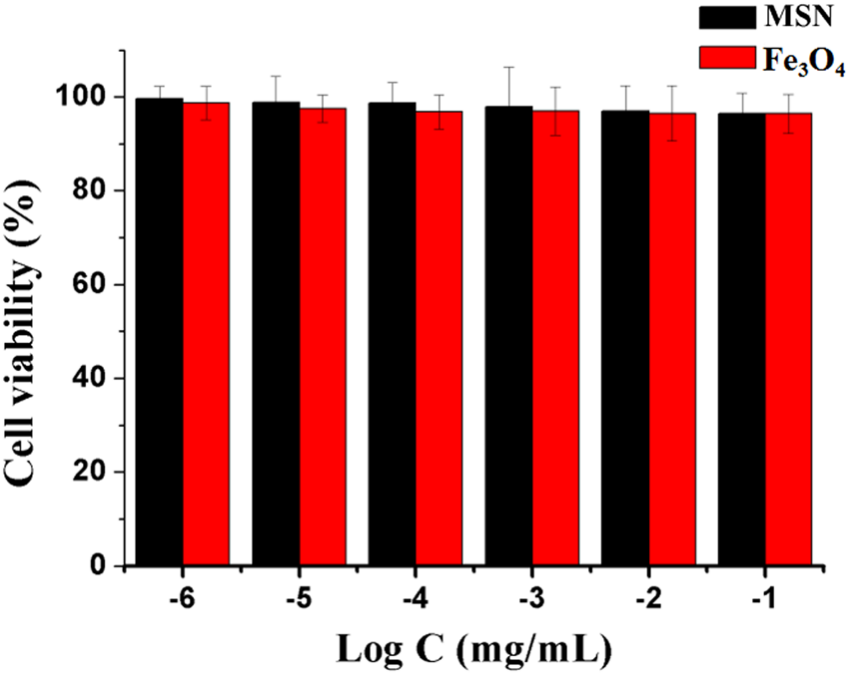
*In vitro* cytotoxicity of MSN and Fe_3_O_4_ incubated with MDA-MB-231 cells after 48 h incubation.

**Figure 7:**
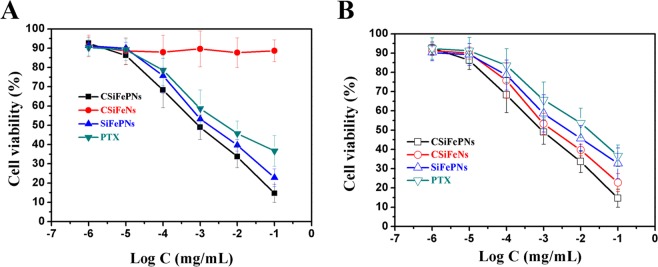
(**A**) *In vitro* cytotoxicity of PTX, SiFePNs, CSiFeNs and CSiFePNs incubated with MDA-MB-231 cells out of alternating magnetic field after 48 h incubation. (**B**) *In vitro* cytotoxicity of PTX, SiFePNs, CSiFeNs and CSiFePNs incubated with MDA-MB-231 cells in alternating magnetic field after 48 h incubation.

The cytotoxicity of nanoparticles was investigated using MTT assay. MSN and Fe_3_O_4_ both show negligible cytotoxicity at the tested concentrations against MDA-MB-231 cells, indicating their good biocompatibility (Fig. 6). Before exposure to AMF, the MDA-MB-231 cells incubated with CSiFeNs show almost negligible toxicity (Fig. 7A). However, CSiFeNs exhibit the IC_50_ value of 3.6 μgL^−1^ after exposure to AMF, indicating that AMF induced the magnetotherapy of CSiFeNs (Fig. 7B). Before and after exposure to AMF, PTX shows almost similar cytotoxicity, indicating that AMF has no significant effects on the cytotoxicity of PTX. It is exciting that the MDA-MB-231 cells incubated with CSiFePNs show the IC_50_ value of 0.8 μgL^−1^, 3.5-fold higher than that of SiFePNs. Moreover, the IC_50_ value of CSiFePNs in AMF is higher than that of CSiFePNs out of AMF. This may be attributed to the increased cellular uptake brought out by proteins on the cell membrane surface^45^. In summary, these results all demonstrate that CSiFePNs make an excellently synergistic therapeutic effect on the MDA-MB-231 cells *in vitro*. The antiproliferative effects of CSiFePNs in AMF are stronger than both that of CSiFeNs irradiated by AMF and CSiFePNs without any magnetic irradiation. The combination method of magnetotherapy and chemotherapy leads to a significant binding inhibition impact on the MDA-MB-231 cells. CSiFePNs can induce cytotoxicity for two reasons. On the one hand, paclitaxel in nanoparticles is one of the causes of cell apoptosis due to the killing effect of paclitaxel on tumor cells. On the other hand, the superparamagnetic Fe_3_O_4_ in the CSiFePNs can perform magnetocaloric transformation under the action of an alternating magnetic field. The magnetocaloric reaction causes the rising temperature of the MDA-MB-231 cells, and then the apoptosis occurs after exceeding the temperature tolerance range of cells^38,49^. These two reasons together lead to apoptosis of tumor cells. The cytotoxicity of CSiFePNs loaded with the chemotherapeutic drugs paclitaxel and Fe_3_O_4_ is much higher than the previously reported cytotoxicity of magnetic nanoparticles containing only Fe_3_O_4_^40^.

On the one hand, the growth inhibition of MDA-MB-231 cells is affected by the accumulation of PTX. With the increase of the accumulation of PTX in MDA-MB-231 cells, their growth inhibition is also improved. CSiFePNs are more likely to enter into the MDA-MB-231 cells since CSiFePNs have the same cell membrane with the MDA-MB-231 cells. Homologous cell membrane enhances the targeting of CSiFePNs to the MDA-MB-231 cells, which can also be recognized by macrophages from being phagocytized. As the cellular uptake of CSiFePNs increases, the drug amounts inside the MDA-MB-231 cells also grow. Thus, the growth inhibition effect of the MDA-MB-231 cells are enhanced, which is consistent with the IC_50_ value of CSiFePNs’ of 0.8 μgL^−1^, 3.5-fold higher than that of SiFePNs. On the other hand, the superparamagnetic ferroferric oxide in CSiFePNs has a magnetocaloric effect^46^. If the paramagnetic and ferromagnetic substances are affected under the action of the external magnetic field, the magnetic moment can be changed from clutter to order. Then, the interaction between the atomic magnetic moment and the external magnetic field is reduced, so is its magnetic entropy. The process of excreting entropy is also a process of exotherm^50^. Therefore, CSiFePNs generate heat under the action of AMF to raise the internal temperature of cells. For temperatures above 40 °C, cancer cells are more sensitive than healthy cells. The biological integrity of the cancer cell membrane and cytoskeleton can be damaged and cancer cells may become apoptotic^51,52^. Therefore, CSiFePNs can kill the MDA-MB-231 cells by magnetic hyperthermia under the action of AMF. In clinical treatment, high concentration of superparamagnetic ferroferric oxide is often required to play a therapeutic role. However, CSiFePNs in this study effectively play an important role in killing MDA-MB-231 cells under the combined effects of chemotherapy and magnetic hyperthermia.

Laser confocal microscopy study was used to demonstrate that MDA-MB-231 cell membranes encapsulating nanoparticles could improve the uptake of nanoparticles by the MDA-MB-231 cells. Figure S3 shows the confocal microscopy images of MDA-MB-231 cells after 2 h incubation with SiFePNs-Coumarin-6 and CSiFePNs-Coumarin-6 at 37 °C. The microscopic image showed that the green fluorescence from CSiFePNs-Coumarin-6 was more and stronger than that from SiFePNs-Coumarin-6 in the cell cytoplasm. This indicated that MDA-MB-231 cell membrane modification could impove penetration of the mesoporous silica nanoparticles into the MDA-MB-231 cells.

## Conclusion

In summary, we prepared a biomimetic nanodrug delivery system with cell membranes to inhibit the growth of MDA-MB-231 cells by using the combination method of chemotherapy and magnetic hyperthermia. In addition, the characterizations of particle size and potential analysis, Nitrogen adsorption-desorption measurement, FT-IR study and Raman spectra analysis indicate that CSiFePNs containing superparamagnetic ferroferric oxide and Paclitaxel coated with MDA-MB-231 cell membranes were prepared successfully. The *in vitro* cytotoxicity tests show that the MDA-MB-231 cells incubated with CSiFePNs obtained the IC_50_ value of 0.8 μgL^−1^, 3.5-fold higher than that of SiFePNs. Higher inhibition rate of tumor cell growth indicates that the combination of chemotherapy and magnetocaloric therapy has great potential application to the treatment of breast cancer. In conclusion, CSiFePNs can serve as a promising biomimetic drug delivery with the targeting of homotypic tumor cells and the magnetocaloric ability for future breast cancer therapy. We believe that our research will also bring new opportunities for tumor cell membranes as vectors to target homologous cells.

## Supplementary information

Supporting information
