# Supplementary material for: Cancer cell membrane-coated mesoporous silica loaded with superparamagnetic ferroferric oxide and Paclitaxel for the combination of Chemo/Magnetocaloric therapy on MDA-MB-231 cells
Source: Sci Rep. 2019 Oct 9;9:14475. doi: 10.1038/s41598-019-51029-8 (PMC6785558; doi:10.1038/s41598-019-51029-8)
Supplement: Supplementary file 1 — Supporting information [file 41598_2019_51029_MOESM1_ESM.docx]

**[Cancer cell membrane-coated mesoporous silica loaded with superparamagnetic ferroferric oxide and Paclitaxel for the combination of Chemo/Magnetocaloric therapy](https://www.sciencedirect.com/science/article/pii/S0142961217304829) on MDA-MB-231 cells**

Defu Cai^1^, Likun Liu^1^, Cuiyan Han^2^, Xiaoxing Ma^2^, Jiayi Qian^2^, Jianwen Zhou^1^, Wenquan Zhu^2^*(Correspondent)


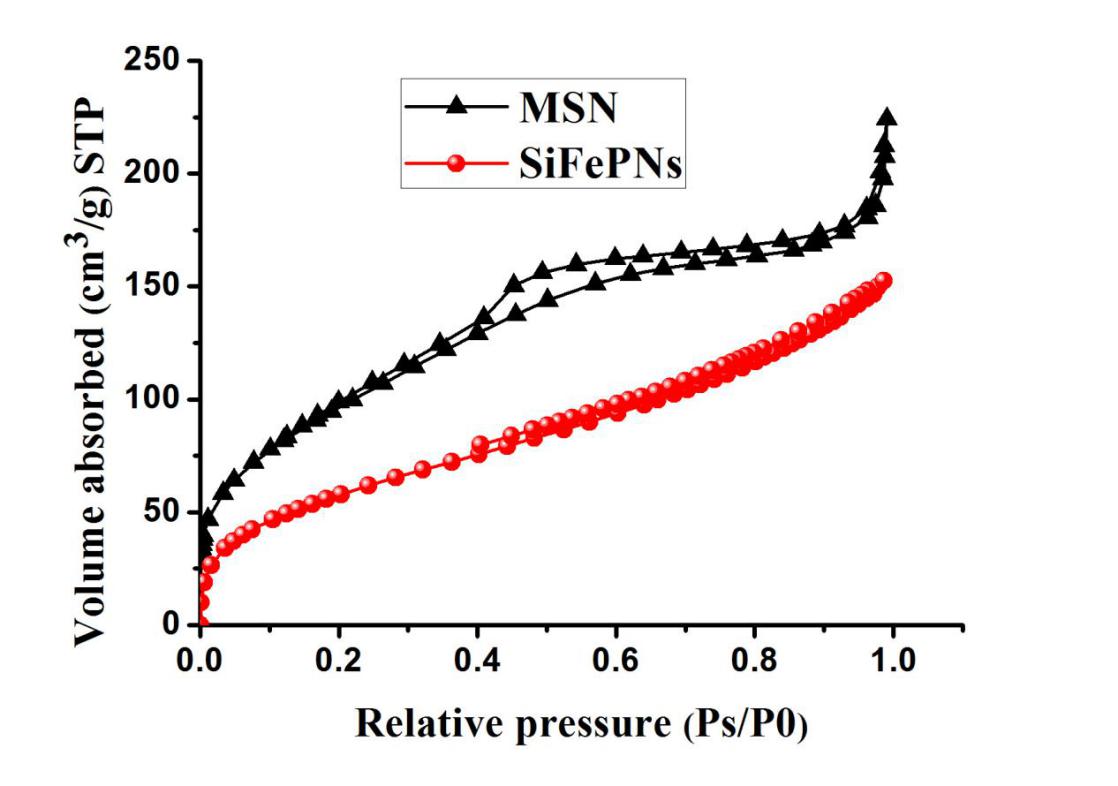


**Figure S1.** Adsorption–desorption isotherms of MSN and SiFePNs.


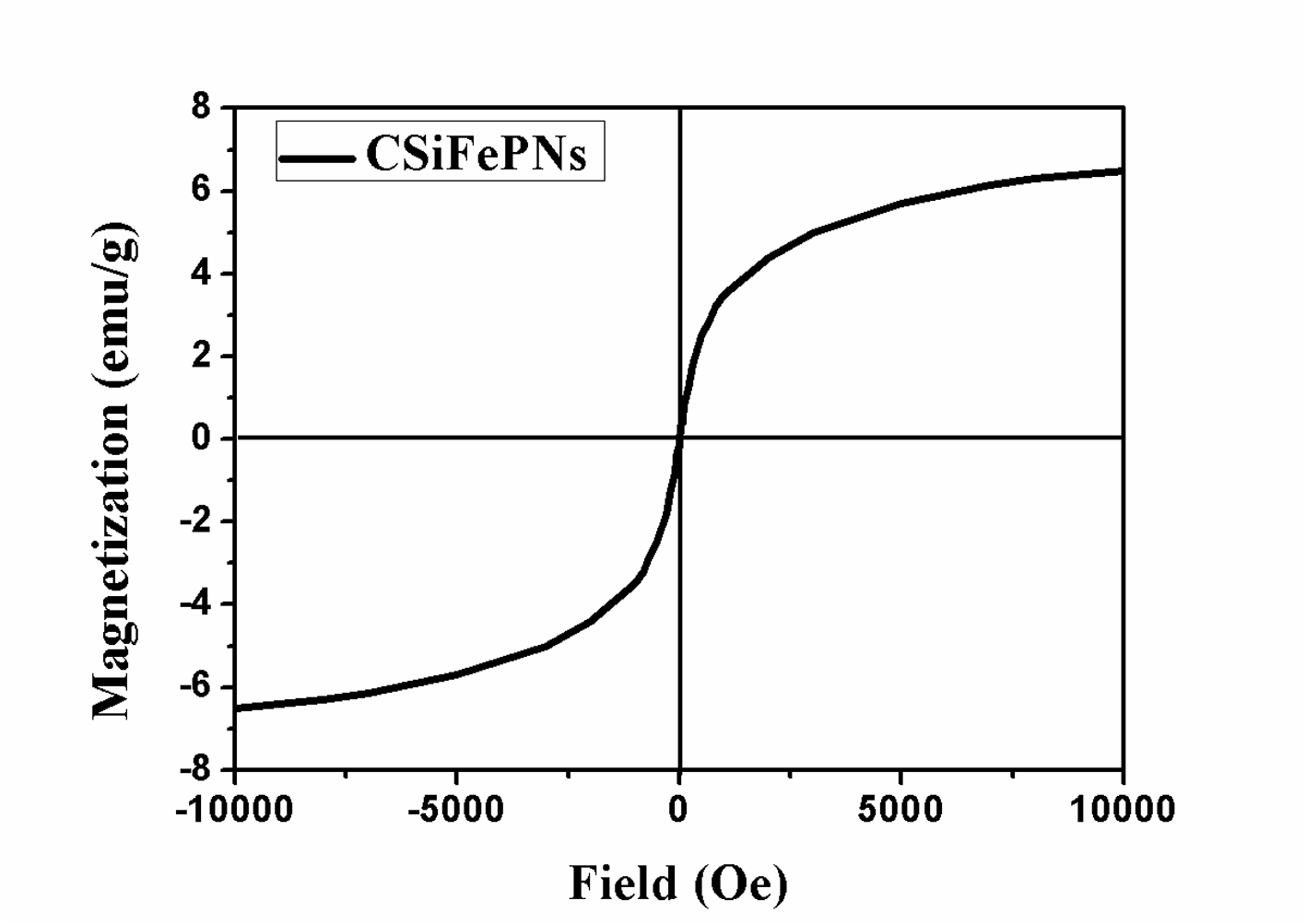


**Figure S2.** Magnetization curve of CSiFePNs**.**

*
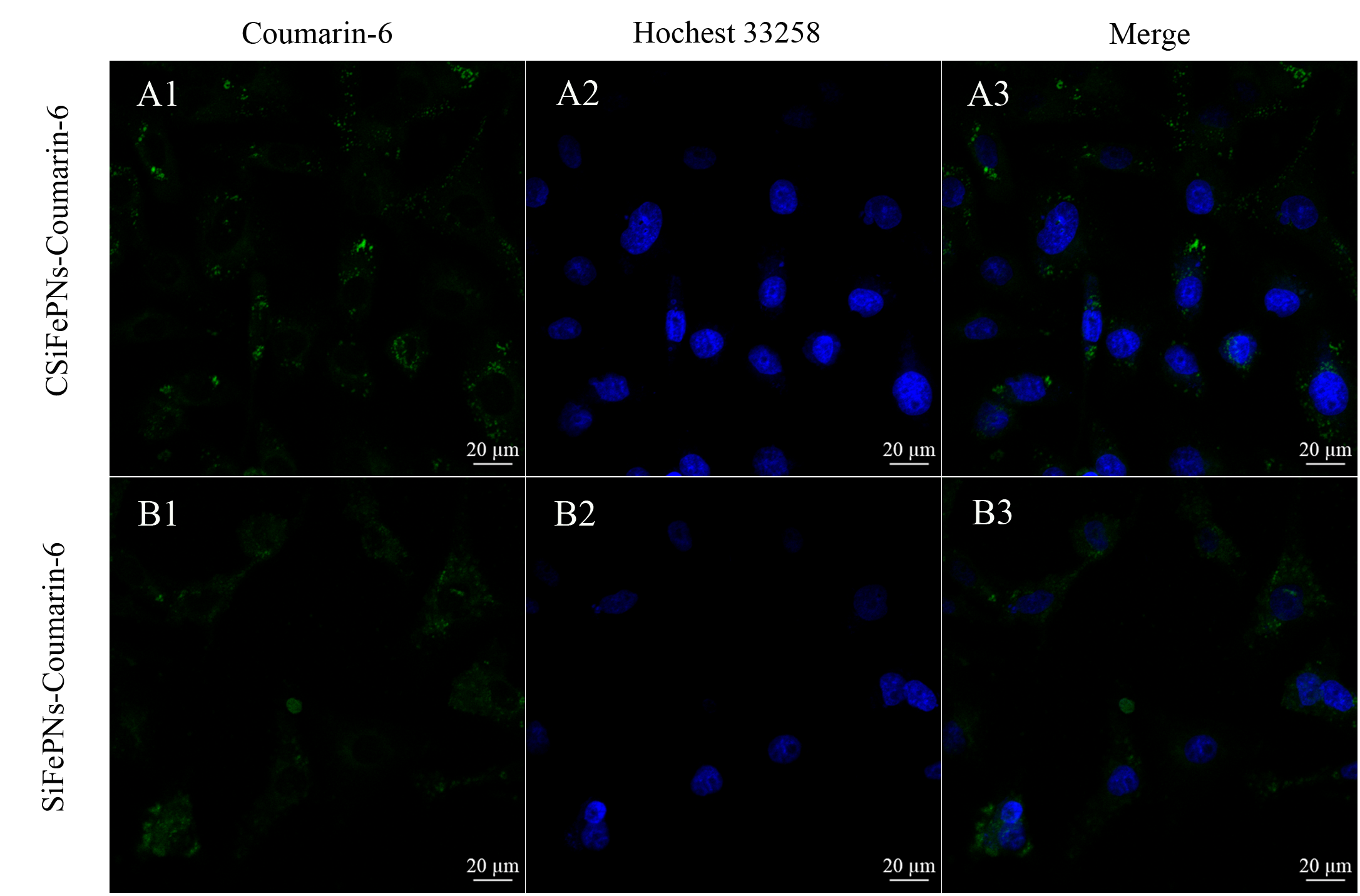
*

**Figure S3** Laser scanning confocal microscopy images of MDA-MB-231 cells incubated with SiFePNs and CSiFePNs at 37 ℃ for 2 h. The images of CSiFePNs-Coumarin-6 (Figure 8A1, A2 and A3), The images of SiFePNs-Coumarin-6 (Figure 8B1, B2 and B3). Green and blue indicate the fluorescence of Coumarin-6 and Hoechst 33258, respectively. Scale bars represent 20 mm.
